# ZnO Nanoparticle/Graphene Hybrid Photodetectors via Laser Fragmentation in Liquid

**DOI:** 10.3390/nano10091648

**Published:** 2020-08-21

**Authors:** Kristin Charipar, Heungsoo Kim, Alberto Piqué, Nicholas Charipar

**Affiliations:** U.S. Naval Research Laboratory, 4555 Overlook Ave., SW, Washington, DC 20375, USA; heungsoo.kim@nrl.navy.mil (H.K.); alberto.pique@nrl.navy.mil (A.P.); nicholas.charipar@nrl.navy.mil (N.C.)

**Keywords:** graphene, laser fragmentation, laser processing, nanoparticles, ultraviolet photodetection, zinc oxide

## Abstract

By combining the enhanced photosensitive properties of zinc oxide nanoparticles and the excellent transport characteristics of graphene, UV-sensitive, solar-blind hybrid optoelectronic devices have been demonstrated. These hybrid devices offer high responsivity and gain, making them well suited for photodetector applications. Here, we report a hybrid ZnO nanoparticle/graphene phototransistor that exhibits a responsivity up to 4 × 10^4^ AW^−1^ and gain of up to 1.3 × 10^5^ with high UV wavelength selectivity. ZnO nanoparticles were synthesized by pulsed laser fragmentation in liquid to attain a simple, efficient, ligand-free method for nanoparticle fabrication. By combining simple fabrication processes with a promising device architecture, highly sensitive ZnO nanoparticle/graphene UV photodetectors were successfully demonstrated.

## 1. Introduction

Optoelectronic devices utilizing graphene have been studied extensively over the past decade, paving the way for the fabrication of thin, lightweight, highly efficient devices. The advantages of graphene in sensor applications are numerous, including high mobility (>10^4^ cm^2^ V^1^s^−1^) [1], optical transparency (~2.3% for monolayer) [2,3], excellent mechanical and chemical stability, and an inherently ultrathin, flexible form factor [3,4]. While a reported absorption of ~2.3% is quite large for monolayer materials [2], it is insufficient for high quantum efficiency optoelectronic devices. Additionally, because the ultrafast exciton lifetime of graphene leads to fast carrier recombination times [5,6], photocurrent development is hindered making graphene alone not ideal for photoconductor applications [6,7]. Nonetheless, by combining photosensitive nanostructures, such as metal oxide semiconductors, with graphene as a transport layer, many enhanced effects are observed [8]. These photodetectors can be tailored to operate in specific spectral ranges depending on the bandgap of the material (e.g., ZnO for ultraviolet detection, PbS for near-infrared detection [7], and Ti_2_O_3_ for mid-infrared detection [9]). 

Because of its wide band gap (~3.3 eV) and high exciton binding energy (~60 meV), zinc oxide (ZnO) is a promising candidate for ultraviolet (UV)-sensitive hybrid photodetectors [8]. In addition, ZnO is radiation-resistant and non-toxic making it an attractive material for wearable sensor technologies. While the inherent mobility of bulk crystalline ZnO is not high (~200 cm^2^ V^−1^s^−1^ at room temperature) [10], its combination with graphene offers an efficient charge transport pathway due to the high mobility of graphene resulting in significant photoconductive gain. Moreover, ZnO has enhanced wavelength selectivity in the UV range, while graphene provides broadband optical transparency, thus enabling solar-blind photodetectors. As a result, photodetectors that combine ZnO and graphene have been studied recently by many research groups. A wide assortment of ZnO structures has been studied for UV photodetection, including nanoparticles [11,12,13,14,15], nanowires [16,17,18,19], and thin films [20,21,22,23]. Because of the unique properties afforded by nanoscale structures, an optimal size is achieved when the ZnO nanostructures approach the Debye length, which is on the order of ~18 nm [24]. At this size scale, the surface depletion effect is maximized, shortening the carrier transit time, leading to photoconductive gain. In addition, by using nanoparticles instead of bulk ZnO thin films, the high surface-to-volume ratio provides a high density of hole trap states for charge transfer into the underlying graphene layer [14].

Many different techniques have been utilized to fabricate ZnO nanostructures for hybrid graphene photodetector applications, such as hydrolysis methods for nanoparticle fabrication [15] and hydrothermal [18] and chemical vapor deposition [19] methods for nanowire synthesis. To simplify the nanostructure fabrication process, we demonstrate the use of pulsed laser fragmentation in liquid (PLFL) as an alternative for nanoparticle generation. This technique relies on ultrafast laser pulses to generate nanoparticles in solution via various physicochemical processes. It offers the advantages of simple experimental set-up, control over size distribution and particle morphology, and the potential to maintain the stoichiometry of the original particle [25]. PLFL of Ag nanoclusters was first demonstrated over two decades ago by Kamat et al. [26], followed by many other research efforts focused primarily on noble metal nanoparticles [27,28]. In recent years, the use of PLFL has extended well beyond Au and Ag to other metals [29], alloys [30], and semiconductors [31], including indium tin oxide [32] and ZnO [33,34]. 

Here, we have demonstrated PLFL for the synthesis of ZnO nanoparticles with a bimodal size distribution (~18 nm and 46 nm). These nanoparticles were integrated into graphene-based hybrid phototransistors, which were then characterized to determine the optical and electrical performance, including wavelength selectivity and responsivity. 

## 2. Materials and Methods 

Phototransistor devices were fabricated using standard wet transfer [35,36,37,38] microfabrication processing techniques. Highly-doped (0.001 Ω·cm–0.005 Ω·cm) Si wafers with a 285 nm thermal oxide layer were laser-diced to 2 cm × 2 cm. The SiO_2_ on the backside of the Si wafer was laser-micromachined to expose the highly conductive Si for device back-gating. Graphene on Cu foils (Graphene Supermarket, Ronkonkoma, NY, USA) were spin-coated with poly(methyl methacrylate, or PMMA (Kayaku Advanced Materials, Westborough, MA, USA, 495 PMMA A2) resulting in a ~600 nm thick layer. The foils were then baked at 100 °C in air for 2 min on a hot plate. The graphene on the backside of the Cu foil was etched by floating the foil on a 10% HNO_3_ solution for 3 min followed by rinsing with deionized water. The Cu was remove by etching in a ferric chloride solution for 2 h. The remaining PMMA/graphene film was then floated on a dilute 2% HCl solution to remove any particulates introduced during the Cu etching process. The film was rinsed in deionized water before wet transfer. The PMMA/graphene film was then transferred onto the SiO_2_/Si substrate and allowed to air dry. A small droplet of PMMA was drop casted onto the surface of the PMMA/graphene to encourage flattening of the film, followed by air drying. The PMMA was then removed with acetone, followed by rinsing in isopropanol and then water. After processing the graphene, source and drain electrodes (5 nm Ti/150 nm Au) were deposited via electron beam evaporation using a shadow mask that was laser-micromachined from a thin (75 µm) polyetherimide sheet. After electrode deposition, isolation lines were laser-micromachined around each device on the chip, yielding active device areas of 2 mm × 1 mm.

**Figure 1:**
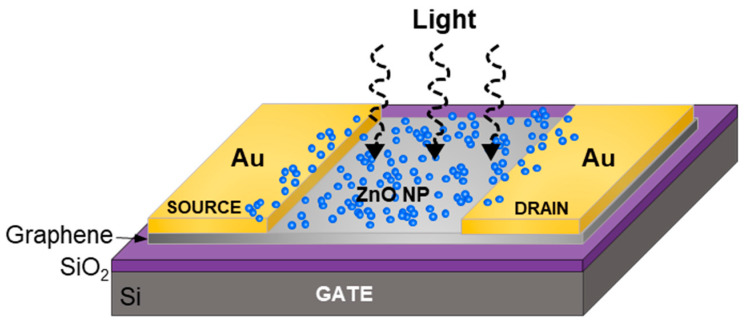
Schematic of the ZnO nanoparticle/graphene phototransistor architecture (not to scale).

ZnO nanoparticles were produced using pulsed laser fragmentation in liquid (PLFL) [25]. ZnO powders were used as received (Millipore Sigma, St. Louis, MO, USA 140 nm avg. diameter) and dispersed in deionized water at 0.1 wt%. PLFL was performed using a pulsed femtosecond laser system (Light Conversion Ltd, Vilnius, Lithuania, Pharos Yb:KGW laser, λ = 1030 nm, 10 kHz, pulse duration ~200 fs). The ZnO particle solution was laser-treated for 1 h at a laser pulse energy of 17 µJ. The laser was focused with a 10 cm focal length lens into a quartz cuvette containing the ZnO/water solution. Because the laser spot was focused into a cuvette containing the ZnO solution, it was difficult to determine an exact fluence as the laser light was absorbed and scattered by the ZnO particles as the beam converged into focus. After PLFL, the water was exchanged for ethanol via centrifugation and decanting. The final solution was sonicated to re-disperse the nanoparticles and break apart any agglomerates. The final ZnO/graphene devices were fabricated by drop-casting the ZnO nanoparticle ethanol solution onto the active graphene area of the previously fabricated phototransistors. The ethanol was allowed to evaporate in air resulting in a film of ZnO nanoparticles across the entire device. ZnO nanoparticles fabricated by PLFL were characterized via scanning electron microscopy (JEOL USA Inc., Peabody, MA, USA, JSM7001F), particle analysis, and photoluminescence measurements (343 nm excitation source) to determine final particle size, distribution, and quality, respectively. Additionally, the optical absorption spectra of ZnO nanoparticle solutions were collected using a UV/Vis spectrophotometer (JASCO Inc., Easton, MD, USA, V670). A schematic of the final phototransistor device with ZnO nanoparticles dispersed on the surface is shown in Figure 1.

The optical properties of the fabricated graphene transistors before nanoparticle deposition were characterized via Raman spectroscopy (WITec Instruments Corp., Knoxville, TN, USA, alpha300 RAS) which revealed the quality of the graphene layer. Optoelectronic characterization was performed using UV illumination that was fiber coupled from a monochromator into a 10× objective, mounted on a probe station. The light intensity was adjusted by a computer-controlled attenuator, maintaining a uniform spot size of ~2 mm for all experiments. Electrical characterization, including the drain and gate sweeps as well as temporal measurements, was conducted using a semiconductor characterization system (Keithley Instruments, Solon, OH, USA, 4200SCS).

## 3. Results and Discussion

### 3.1. Pulsed Laser Fragmentation in Liquid (PLFL)

**Figure 2:**
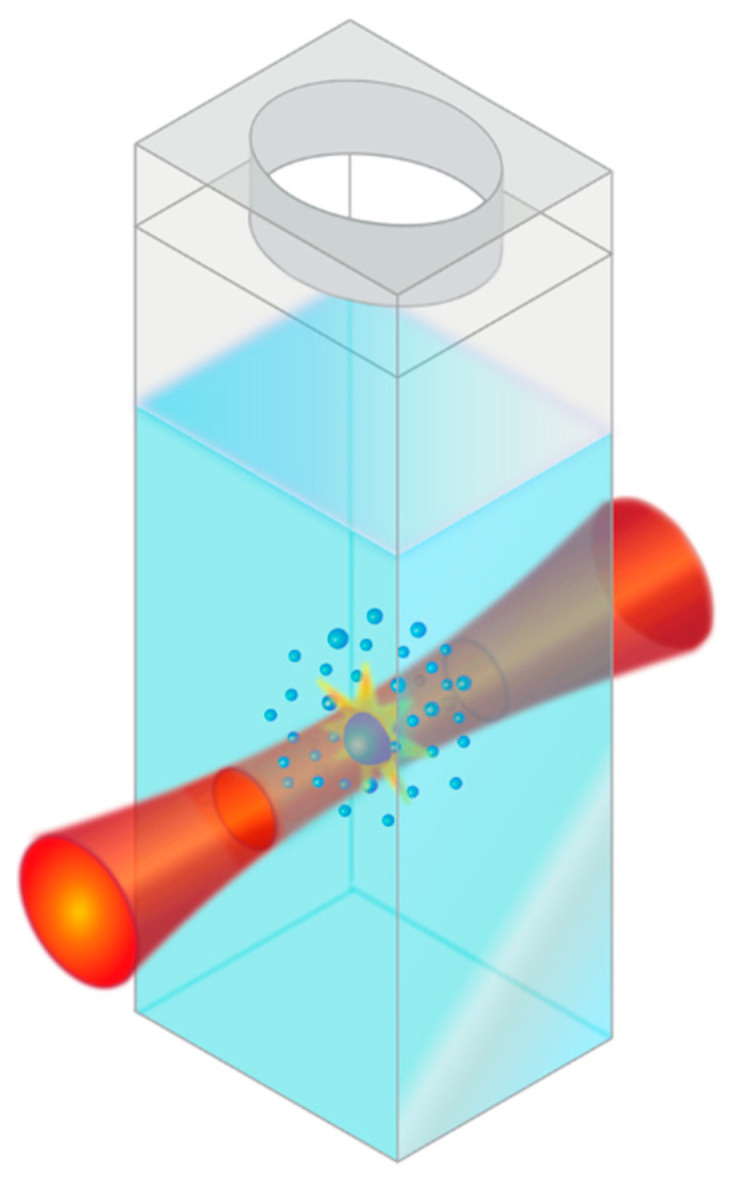
Schematic of the pulsed laser fragmentation in liquid (PLFL) process, where an aqueous solution of ZnO particles is irradiated with a femtosecond IR laser to synthesize smaller ZnO nanoparticles (not to scale).

Because of the unique properties afforded by nanoscale materials, different methods have been developed for simple and efficient fabrication, including wet chemical synthesis [12], sol gel [39], thermal vaporization [40], and pyrolytic reactions [41]. Chemical synthesis methods are often time-consuming, complex multi-step processes, involving a variety of potentially hazardous materials and solvents. Additionally, these chemical synthesis methods often require the use of ligands either during or after fabrication of nanoparticles [42], which can affect nanoparticle packing and electrical transport, ultimately impacting device performance. Alternatively, a ligand-free synthesis technique that has been widely studied is pulsed laser ablation in liquid (PLAL), which relies on laser–matter interactions for the generation of nanostructures typically from bulk materials [43]. There has been much research conducted on the generation of ZnO nanoparticles via PLAL; however, typical experiments involve the use of either a solid Zn or a ZnO target submerged in a liquid medium [43,44,45,46,47]. In this work, we begin with a ZnO particle powder dispersed in water and use PLFL to create smaller, more uniform nanoparticles. While PLAL is performed using a solid target material, PLFL relies on micro- or nano-sized particles suspended in liquid, which is shown schematically in Figure 2. Similar to PLAL, the resulting size and shape of the particles produced by PLFL can be controlled via pulse energy, pulse duration, and the initial material properties of the target material.

The mechanisms responsible for nanoparticle formation via PLFL and the effect that initial size, concentration and material have on the resulting particle size have been studied extensively [25,26,27,28,29,30,31,32,33,34]. However, the exact mechanisms responsible for nanoparticle formation via PLFL are not entirely understood. Nonetheless, two mechanisms are often used to explain the formation of smaller particles, including photothermal evaporation and Coulombic explosion [25]. During photothermal evaporation, the laser energy is absorbed by the particle, causing surface evaporation when the boiling point of the material is exceeded [28,31]. When the vaporized species cool, they condense into smaller particles. During Coulombic explosion, electrons are ejected from the original particle, generating ionized nanoparticles. These particles then undergo additional fragmentation because of electrical charge repulsion [48]. These two mechanisms can occur independently or can compete depending on material properties and operating conditions (laser pulse duration and laser fluence, to name a few). Additionally, PLFL can often be accompanied with some degree of simultaneous laser melting. During the PLFL process, laser attenuation in the liquid can create a fluence gradient, where a portion of the liquid experiences a low fluence regime that results in laser melting of the particles. Thus, the resulting nanoparticle size is often a complex balance between the laser fragmentation process which reduces particle size with a laser melting process which can cause the produced nanoparticles to coalesce and grow [25].

There are several advantages to this technique, including simple experimental set-up and the ability to maintain complex stoichiometries with narrow particle size distributions [49]. The mechanism of particle formation allows for nanoparticle surfaces that are ligand-free [25]. While traditional solution-based nanoparticle synthesis methods often involve the use of ligands either during or after synthesis [42,50], PLFL offers a ligand-free fabrication method [25]. Solution-based chemistry techniques can leave insulating surface chemistries on the nanoparticle that can be time consuming to remove before device integration. This is important because ligands can interfere with optoelectronic device performance and efficiency by inhibiting charge transport and preventing the close-packing of particles [51,52]. Additionally, PLFL offers a method to produce bimodal size distributions, further enhancing particle packing which can improve performance [53]. 

**Figure 3:**
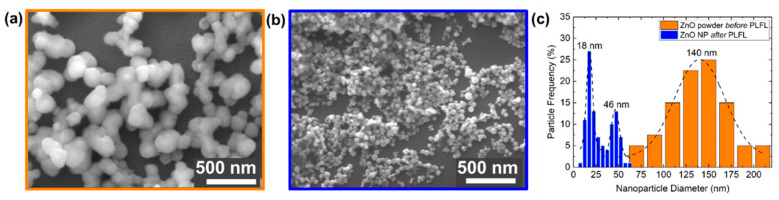
SEM micrographs of ZnO nanoparticles (NPs) (**a**) before and (**b**) after pulsed laser fragmentation in liquid (PLFL) processing; (**c**) ZnO nanoparticle size distribution both before and after PLFL.

Both the original ZnO powder particles before PLFL and the resulting smaller nanoparticles after PLFL can be seen in Figure 3a,b, respectively. The largest particles observed after laser processing were ~65 nm and represent a very small fraction (<1%) of the overall particle count. During laser processing, the completion of the fragmentation process was determined via optical scattering. The laser-processed nanoparticles resulted in a bimodal size distribution (Figure 3c), where a portion of the particles were ~18 nm and another portion was ~46 nm, while the original ZnO particles showed a uniform distribution centered around ~140 nm. This bimodal distribution of nanoparticles as a result of PLFL [33] is of interest because the smaller particles are close to the Debye length for ZnO, which is on the order of ~18 nm. At this length scale, the depletion layer on the ZnO nanoparticle surface is enhanced, which minimizes the photodetector response time while maximizing responsivity. The bimodal distribution (r_small_/r_large_ ≈ 0.42) is potentially advantageous for the final device design because a more efficient packing factor becomes possible compared to the unimodal particle distribution. For bimodal spheres, ideal packing can be efficiently achieved up to a particle size ratio of ~0.41 [53], where the smaller particle simply fits into the interstitial spaces between the larger particles. Additionally, it has been shown that UV absorption in ZnO nanoparticles is dependent on nanoparticle diameter, where absorption increases as the particle diameter increases, peaking at 40 nm and then decreasing as the particle size increases beyond 40 nm [54]. 

To give insight into the quality and defect content of the generated nanoparticles, photoluminescence measurements (λ = 343 nm) were conducted. The photoluminescence of ZnO nanoparticles has been studied extensively and typically reveals two distinct emission bands, one in the UV and one in the visible spectrum. The emission peak observed in the UV region is a result of near-band-edge emission which is mediated through exciton–exciton interactions. As the particle size decreases, the fluorescence is blue-shifted due to an increase in transition energy [55,56]. The second photoluminescence peak for ZnO is commonly observed as a green emission and is most likely due to deep level emission in the band gap through electron-hole recombination. This green emission peak is often broad and weak compared to the UV peak, with emissions lines reported from 510–583 nm [57]; however, other visible emission has been observed, including blue, yellow, violet, and red. The cause of these different visible emission peaks is still controversial, but is attributed to intrinsic defects such as Zn interstitials, oxygen vacancies, and the formation of free carriers [56,58,59]. It has been shown that green emission can be suppressed by coating the ZnO nanoparticle surface with surfactants, suggesting that surface defects are responsible [57,60]. Specifically, the mechanism responsible for green emission is often partially attributed to single ionized oxygen vacancies [56,60].

**Figure 4:**
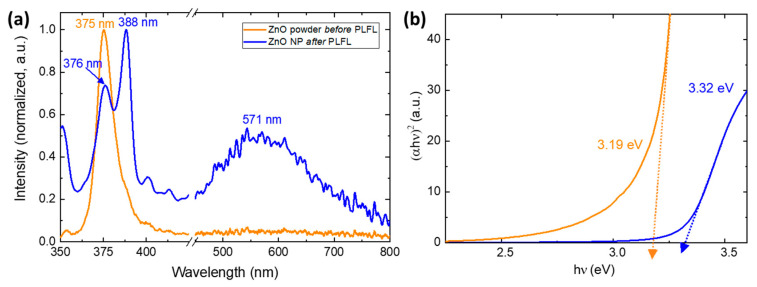
(**a**) Photoluminescence spectra and (**b**) Tauc plots for the ZnO nanoparticles both before (orange lines) and after (blue lines) PLFL processing.

The as-received ZnO powders, which can be seen in Figure 4a, show a strong, narrow UV emission band at 375 nm and no discernible green emission, indicating high quality, low surface defect particles. A second UV emission peak is then observed in the photoluminescence spectra of ZnO nanoparticles generated via PLFL, which can also be seen in Figure 4a. The size of these particles is unlikely to directly affect the UV peak emission position, as quantum confinement effects are not observed at these scales because the Bohr radius of ZnO is significantly smaller at ~2.34 nm [57]. This second UV peak, observed at ~388 nm, can be attributed to either band-edge exciton emission or energy transitions involving Zn interstitials [57]. These UV emission peaks are both strong and narrow, but a broad, weaker peak seen at 571 nm indicates defect states on the ZnO nanoparticle surfaces. The emission characteristics of ZnO typically exhibit stronger UV peaks with structures of larger size with better crystalline quality, while smaller, more defective surface states show higher visible emission. While the exact origin of the green defect emission in ZnO remains contentious and poorly understood, there are several processing parameters that can be adjusted to control this defect emission, including solvent choice [61,62].

In addition to the insight into the quality and defect density induced in the nanoparticles during laser processing, the optical transmission spectra of the ZnO nanoparticle solutions were collected to help understand the effect of laser-processing on the particles optical bandgap, which is shown in Figure 4b before and after PLFL. 

### 3.2. ZnO Nanoparticle/Graphene Phototransistors

**Figure 5:**
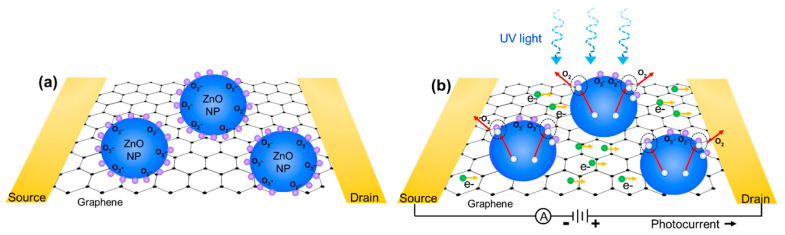
Schematic of photoconduction mechanism for ZnO nanoparticle/graphene photodetectors (**a**) without and (**b**) with UV illumination (not to scale).

The mechanism of photoconduction in ZnO nanoparticle/graphene phototransistors is schematically illustrated in Figure 5. In the absence of UV light, oxygen molecules adsorb onto the ZnO nanoparticle surface and capture free electrons which form oxygen ions, creating a low conductivity depletion zone on the surface of the ZnO nanoparticles. When illuminated with UV light with energy higher than the bandgap of ZnO (~3.3 eV), electron-hole pairs are generated with the holes crossing the depletion layer and traveling to the surface of the ZnO nanoparticle. These holes recombine with negatively charged oxygen ions which results in the desorption of neutral oxygen molecules [15]. The remaining unpaired electrons in the conduction band of the ZnO nanoparticles transfer to the graphene layer, where they move to the drain electrode as a result of an applied source–drain voltage potential, resulting in a change in channel resistance. It is known that the size of the nanoparticle affects the performance of the phototransistor, where ZnO nanoparticles, close to or smaller than the Debye length (~18 nm), allow for a high density of trapped hole states on the surface, providing substantial photoconductive gain. Thus, by combining the advantageous surface depletion zone achieved with ZnO nanoparticles and a high mobility of graphene layers, enhanced responsivity and gain can be achieved in a hybrid photodetector.

**Figure 6:**
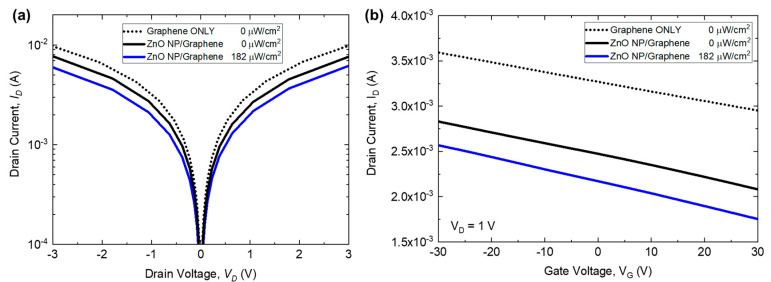
Electrical transport properties of both the ZnO/graphene phototransistors and the graphene-only phototransistors. Photocurrent as a function of (**a**) drain voltage and (**b**) the applied gate voltage at a drain voltage, V_D_ of 1V.

To understand the photoresponse of the devices, electrical transport properties were measured with UV illumination (λ = 365 nm), from 34 µW/cm^2^ up to 1.4 mW/cm^2^. Drain current as a function of drain voltage can be seen in Figure 6a, where different illumination conditions are plotted. The I–V characterization shows a bipolar behavior as a function of drain voltage. By applying a gate voltage to the transistor, an electric field is produced which can enhance the device response. Additionally, drain current as a function of gate voltage can be seen in Figure 6b.

The responsivity and gain as a function of incident UV power were measured to determine the performance of the phototransistors. The responsivity of the devices is the ratio of the measured photocurrent to the UV illumination, where:

(1) $$R=\frac{I_{ph}-I_{dark}}{P}=\frac{\Delta I}{P}$$

**Figure 7:**
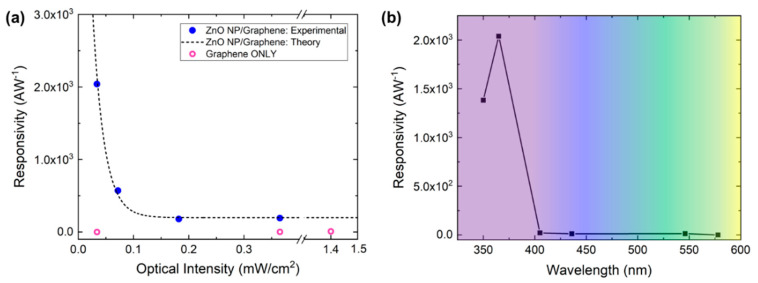
(**a**) Responsivity as a function of optical intensity for both ZnO nanoparticle/graphene devices (solid blue circles) and graphene only devices (open pink circles). Dashed black line represents a simulated fit. (**b**) Responsivity as a function of incident wavelength for ZnO nanoparticle/graphene devices.

Here, *I_ph_* and *I_dark_* are the induced photocurrent under UV illumination and the dark current, respectively. The responsivity at a drain current of V_D_ = 5 V with no gate voltage applied can be seen in Figure 7a. As the incident power approaches zero, the responsivity can be extrapolated and rises to a maximum of ~4 × 10^4^ AW^−1^, where experimentally at *P* = 34 µW/cm^2^, the responsivity is measured to be 2 × 10^3^ AW^−1^. The photoconductive gain *G* can be described as the ratio between the number of electrons collected per unit time and the number of absorbed photons per unit time [12,19]:

(2) $$G=\frac{\Delta I}{qF}=\frac{\Delta I}{P}\cdot \frac{hc}{e\lambda }=R\cdot \frac{hc}{e\lambda }$$

where *h* is Planck’s constant, *c* is the speed of light, *e* is electron charge, and *λ* is incident wavelength. By fitting the available data, the maximum gain achieved as *P* approaches zero is G_max_ ≈ 1.3 × 10^5^ at V_D_ = 5 V with no applied gate voltage. The gain of the ZnO nanoparticle/graphene phototransistor is a function of the applied optical intensity. The number of electron-hole pairs generated is directly related to the optical intensity applied, where with a higher intensity, more hole trap states are filled at the surface eventually reaching a surface saturation state. Once this occurs, the electron-hole pairs generated do not aid in charge transfer into the graphene layer, thus limiting the efficiency of the hybrid device [15].

In addition to responsivity and gain, the wavelength selectivity of the photodetector is critical because it determines the spectral range. The spectral dependence of responsivity for ZnO nanoparticle/graphene devices can be seen in Figure 7b, where the photoresponse of the phototransistors was measured at various wavelengths, chosen to correspond with the experimental UV source (Hg lamp) emission lines. Since the bandgap of the ZnO nanoparticles was measured to be ~3.32 eV, large photocurrent generation at a wavelength of 365 nm was expected, whereas visible light does not provide enough energy to cause electron excitation to the conduction band [18]. Above ~400 nm, there is no discernable photoresponse in the visible range, making these detectors solar-blind. 

**Figure 8:**
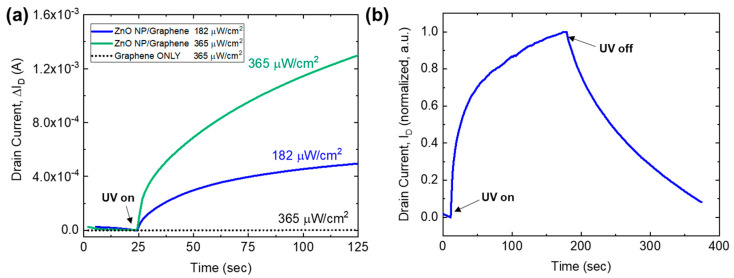
Temporal photoresponse of ZnO nanoparticle/graphene devices: (**a**) ZnO nanoparticle/graphene device compared with the graphene only device at 182 µW/cm^2^ and 365 µW/cm^2^; (**b**) optical intensity of 182 µW/cm^2^ showing the rise and fall as intensity is turned both on and off, respectively.

In order to understand the time-varying behavior of the phototransistors, the photocurrent response was measured as a function of time as the devices were exposed to UV illumination and then subsequently turned off. These temporal measurements were conducted at a drain voltage of V_D_ = 1 V, with no applied gate voltage (V_G_ = 0 V). The graphene-only phototransistors show no photoresponse when illuminated at 365 µW/cm^2^ over a time scale of several hundred seconds, which can be seen in Figure 8a. Other research efforts have demonstrated graphene photoresponse, but the time scale for these changes typically occurs on the order of tens of minutes, so any photoresponse observed in the ZnO nanoparticle/graphene phototransistors can be attributed to photon absorption by the ZnO nanoparticles [14]. The temporal response of the ZnO nanoparticle/graphene detectors at both 182 and 365 µW/cm^2^ can also be seen in Figure 8a, where the change in drain current increases from 500 µA to greater than 1.3 mA. The photoresponse behavior at 182 µW/cm^2^ can be seen in Figure 8b where the UV illumination was turned on and the device was allowed to equilibrate, followed by a recovery time after the illumination was turned off (at ~175 s). A sharp increase in photocurrent was observed once the UV light is turned on, followed by recovery on the order of tens of seconds. 

Both the responsivity and the response times of these ZnO nanoparticle/graphene photodetectors can be optimized by adjusting several experimental parameters. It is well known that the nanoparticle layer is strongly dependent on particle packing in its ability to absorb light and efficiently transfer electrons into the underlying graphene layer [63]. Thus, future studies should focus on controlling the nanoparticle packing factor in an effort to increase the photoresponse and reduce switching speeds. Additionally, the transistor design could be optimized, including making the active area narrower (< mm) in size, which would reduce the possibility for defects introduced during the fabrication process. Because the PLFL process can be controlled via operating parameters, the synthesis of nanoparticles could be optimized to yield even smaller particles. 

## 4. Conclusions

Hybrid ZnO nanoparticle/graphene phototransistors were demonstrated, exhibiting a responsivity of up to 4 × 10^4^ AW^−1^ with a maximum gain of 1.3 × 10^5^ and superior spectral selectivity below 400 nm, making them ideal solar-blind UV photodetectors. We have demonstrated the use of pulsed laser fragmentation in liquid (PLFL) as a simple, ligand-free alternative to traditional nanoparticle synthesis techniques for the fabrication of ZnO nanoparticles.
